# Editorial: Ear-Centered Sensing: From Sensing Principles to Research and Clinical Devices

**DOI:** 10.3389/fnins.2019.01437

**Published:** 2020-01-17

**Authors:** Martin G. Bleichner, Preben Kidmose, Jérémie Voix

**Affiliations:** ^1^Department of Psychology, University of Oldenburg, Oldenburg, Germany; ^2^Department of Engineering, Faculty of Science and Technology, Aarhus University, Aarhus, Denmark; ^3^École de Technologie Supérieure, Montreal, QC, Canada

**Keywords:** ear-centered sensing, bio-physiological monitoring, real-life sensing, wearable sensing, ear-EEG

The human ears are an attractive location for bio-signal acquisition. Heart rate, respiratory rate, skin conductance, eye blink, and eye motion signals, as well as the electrical activity from muscles and the brain can be recorded from the ear. Moreover, the ears provide a discreet and natural anchoring point for placing the necessary wearable hardware, thereby reducing the visibility of integrated devices. In this Research Topic, we define ear-centered sensing as monitoring physiological signals with sensors located in the earcanal (intra-aural), in the pinna, or around the ear (circum-aural). Ear-centered sensing allows data recording over extended periods of time in everyday situations with little disturbance for the users. As the ear is an unconventional place for monitoring these physiological measures, it is necessary to gain a better understanding of the signals and to characterize the signals relative to the conventional measurement methods.

## 1. Sensing

Previous research has shown that a large variety of brain signals can be recorded from electrodes in or around the ears. A number of studies have compared ear-EEG with conventional scalp EEG and estimated the signal loss. In this Research Topic, Kappel et al. show how forward models can estimate to which neural sources the ear-electrodes are most sensitive. They extended the classical head model by including a more precise description of the external ear anatomy. This allows them to compute the sensitivity of in-ear electrodes to cortical sources. This work will help to design better electrode configurations for specific neural sources of interest. While Kappel et al. show that ear-electrodes are most sensitive to sources in the temporal lobe, Garret et al. extend this view and show that also far away sources, i.e., originating from the brain stem can be captured with around the ear-EEG. Finally, Choi and Hwang discuss the role of the reference electrode for ear-EEG recordings. They point toward the fact that the choice of the reference electrode plays an important role in optimally capturing the neural signal of interest. The choice of reference is crucial for the future developments of ear-EEG. For true ear-EEG, ground, active and reference electrode all need to be close to or in the ear. This local setup has an influence on the signals and the amplitudes that can be recorded. Also the choice of the reference site will have an influence on hardware design. A reference electrode contralateral to the recording electrode requires that the electrode on both ears are physically interconnected with an electrical wire.

## 2. Applications

Besides gaining a better understanding of the sensitivity of ear electrodes, a more pragmatic stance can be taken to see in how far ear-EEG in its current form can already be used for different applications. Merrill et al. suggest an authentication procedure based on in-ear EEG. The possibilities to monitor brain activity for extended periods of time using ear-EEG is shown for driving applications. Wascher et al. show that ear-EEG provides information about the mental state of a car driver. A third line of application is the integration of biosensors into hearing devices (i.e., hearing aids or cochlear implants). Nogueira et al. discuss the possibilities of using ear-EEG to decode the direction of auditory attention. Finally, Favre-Félix et al. discuss how ear-EOG (electrooculography) can be used to estimate the direction of attention based on the eye gaze.

## 3. Future Direction

Ear centered sensing has a great potential for integrating biosignals, and in many applications it may be advantageous to combine a plurality of sensor modalities. The combination of physical measurements such as motion, temperature and moisture, and electrophysiological measurements, such as electroencephalography (EEG), electrocardiography (ECG), electromyography (EMG), electrooculography (EOG), and electrodermal activity (EDA), for example, integrated over long time periods, will help to gain a better understanding of psycho-physiological processes. Ear-centered sensing is therefore of interest for scientific, diagnostic and therapeutic purposes and we believe that it will play a significant role in future mobile health applications.

For the sensing of physiological signals over extended periods of time dedicated sensor and amplifier technology is needed that is convenient to use, robust, and reliable. People wearing these sensors should not be restricted in their activities. Hence, for long-term usage sensor and amplifier technology need to be unobtrusive in every aspect: the materials need to be bio-compatible, adjust to the individual's anatomy and be comfortable to wear. They need to be sufficiently robust to allow for continued usage and self-fitting, and they need to be small and inconspicuous.

The electronic instrumentation, including bio-signal amplifiers, analog-to-digital converters, means for signal processing and wireless transmission need to be sufficiently small and light-weight to be placed at the ear together with the sensors. The electronics need to be low-power so that it can be supplied from small batteries or fuel cells, or by harvesting energy from the body or the environment. In ear-centered sensing the electrode distances are typically very small, and often the electrical impedance of the electrode-body interfaces are high, and in consequence there are challenging requirements to the performance of the biosignal amplifier in terms of ultra low noise floor, high common-mode rejection, ultra-high input impedance and high common range.

Finally, the signals need to be processed and interpreted in real-time. This poses new challenges in building systems and devices that can reliably discriminate signals from artifacts when there is little or no control over the recording environment.

## Author Contributions

All authors have participated in writing this editorial and approved it for publication.

### Conflict of Interest

The authors declare that the research was conducted in the absence of any commercial or financial relationships that could be construed as a potential conflict of interest.

